# Urinary hippuric acid level as a biological indicator of toluene exposure on batik workers

**DOI:** 10.1016/j.heliyon.2021.e07775

**Published:** 2021-08-12

**Authors:** Katharina Oginawati, Annisa Artsani Hanif Anka, Septian Hadi Susetyo, Sri Awalia Febriana, Ikeu Tanziha, Cita Rosita Sigit Prakoeswa

**Affiliations:** a Study Program of Environmental Engineering, Faculty of Civil and Environmental Engineering, Institut Teknologi Bandung, Bandung, Indonesia; b Department of Dermatology and Venereology, Faculty of Medicine, Universitas Gadjah Mada, Yogyakarta, Indonesia; c Department of Community Nutrition, Faculty of Human Ecology, Institut Pertanian Bogor, Bogor, Indonesia; d Department of Dermatology and Venereology, Universitas Airlangga, Surabaya, Indonesia

**Keywords:** Hippuric acid, Batik industry, Toluene exposure, Biomarker, Batik wax

## Abstract

Urinary hippuric acid (uHA) is one of the biomonitoring parameters of toxic organic exposure, such as toluene. Repeated exposure to a low concentration of toluene may lead to chronic effects such as central nervous system damage. In the preliminary study, toluene was found in the batik industry's working area in Yogyakarta, Indonesia. This study aims to assess the health condition of batik workers to toluene exposure based on the concentration of uHA. There were 52 respondents divided into 2 groups: the batik workers as the exposed group (30 respondents) and the non-batik workers as the control group (22 respondents). The urine sample was taken from batik workers at the end of the shift, and uHA was measured using spectrophotometry. The uHA value was corrected with urinary creatinine. The results showed that the average uHA concentration of batik workers was higher than that of the control group. The result also showed that workers with more than 2 years working period, writing workers, and stamping workers give an Odds Ratio (OR) of 6.43, 6.75, and 9.00 respectively on having a higher concentration of uHA. Workers with toluene exposure symptoms were also having a higher concentration of uHA than workers without any symptoms.

## 1 Introduction

Batik is one of Indonesia's cultural heritage, acknowledged by The United Nations Educational, Scientific, and Cultural Organization (UNESCO). It is not only as a product but also as a symbol of Indonesian activities [1]. Its common usage makes batik industries are emerging in every region in Indonesia, particularly in Java Island such as Pekalongan, Cirebon, and Yogyakarta. Most of Indonesia's batik industries are home-scale enterprises where the industries are in the same area as the owner's house and run without proper health and safety management system.

The primary material needed for making batik is batik wax, mainly made from paraffin. During all the processes, the batik wax is heated at a certain temperature. The paraffin has a particular viscosity and could be applied easily to develop an excellent stroke [2]. Because of the continuous heating, the process generates batik wax fume into the working area and exposes batik workers. Another step on batik production also requires a heating process to remove the batik wax from the cloth, and this process also generates hot vapor that might be exposed and give a health risk to the workers. Studies on batik wax fume exposure to batik workers show an increase in clinical risk on pulmonary and heart function and a high prevalence of vertigo [3, 4, 5].

Besides the clinical assessment, the other parameter that can be used in health risk assessment is determining the exposure effect on the biochemical changes. Biomarkers are biological molecules found in organ tissues, blood and other body fluids that are signs of normal or abnormal body processes that indicate the condition of a disease. Biomarkers are used to see signs or the body's response to exposure to a pollutant substance [6]. The widely used biological indicator that represent an indication of toluene exposure is urinary hippuric acid (uHA). Toluene will be metabolized primarily into benzoic acid and conjugated with glycine to produce hippuric acid excreted in urine [7, 8]. The uHA is also a natural metabolite found in human urine without toluene exposure with a concentration of about 180–343 mg/g creatinine [9, 10]. The natural source of uHA is mainly from the intake of a phenolic compound that is abundant in berries fruit, chlorogenic and quinic acid in coffee and tea, and benzoic acid in food preservatives [11, 12, 13]. Despite the lack of specificity of uHA, assessment on industrial workers showed a significant correlation between uHA concentration with toluene exposure and neurophysiological symptoms so that the uHA is still widely used [14, 15, 16].

Batik workers mostly work in the indoor area to increase the exposure risk as a toluene concentration indoor is much higher than in the outdoor area [17, 18, 19]. Based on animal studies, toluene exposure can lead to nerve cell morphological disruption, neurobehavioral alteration, and brain transmission signaling inhibition [20, 21, 22, 23]. In humans, toluene exposure may cause liver and kidney dysfunction, central nervous system (CNS) damage, and DNA alteration [24, 25]. In pregnant women, the toluene absorbed into the mother's body will be transported into the fetus, and the concentration on the fetus will be higher compared to the mother's body. This can lead to the baby's early birth, and the child will develop a neurobehavioral and graphomotor disorder when the child is 4–5 years old [26].

Studies on health risk assessment on batik workers in Indonesia are mainly on the effect that has been developed into disease symptoms. More evidence is required regarding toluene exposure and its correlation with worker's health on biochemical changes level. This study aimed to describe batik workers' health conditions based on the toluene exposure symptoms, determine and evaluate uHA concentration on batik workers, also determine the risk factor of having a high level of hippuric acid. This study result can be used as a screening and preventive action of batik workers' health effects.

## 2 Materials and methods

A cross-sectional epidemiological study was conducted from August to October 2020 in three different batik industries in Kulon Progo, Special Region of Yogyakarta, Indonesia. The Ethics Committee has approved this research of Padjajaran University, Bandung, Indonesia.

### 2.1 Study population and sample

A total of 52 respondents were divided into 2 groups: the exposed and control group. The exposed group's inclusion criteria were agreed to participate and signed informed consent, batik workers who work in the production area, have been working for more than 3 months, and 18 years older. Meanwhile, the control group is a resident and an administration staff in the batik industry. Respondents exposed to toluene outside working hours, having liver disease, and consuming medication that might affect toluene metabolism were excluded from this study.

### 2.2 Questionnaire and interview

The questionnaire was done by interviewing each respondent face-to-face before urine sampling. The questionnaire consists of five main parts: individual information, working history, medical history (disease history, medication and medical treatment by a doctor), lifestyle (daily activities such smoking, food and supplement and others), health and safety environment (HSE), and toluene exposure symptoms, both acute and chronic symptoms. For the control group, the working history and HSE part were excluded.

### 2.3 Inhaled toluene exposure

Toluene exposure was measured by collecting inhaled air using a personal sampler pump and charcoal sorbent tube (Anasorb® CSC; SKC). The sampling duration was 3 hours long in the first period (09.00–12.00) or the second period (13.00–16.00) depends on the worker's activities on the sampling day. The toluene concentration and inhaled air characterization then measured using Gas Chromatography-Mass Spectrometry (Shimadzu GCMS-QP2010S) with Rtx-5 column (Shimadzu, 30 m x inner diameter 0.25 mm, film thickness 0.25 μm).

### 2.4 Urine collection

The urine sample was taken by grab sampling at the end of the shift using a 50 mL polystyrene bottle with a minimum volume was 15 ml. The sample was then stored and transported at 4 °C to the laboratory without any additional preservatives and should be tested within 5 days after sampling.

### 2.5 Hippuric acid measurement

Urinary hippuric acid was measured using UV-Vis Spectrophotometry based on the standard methods of NIOSH 8300 Issue 2 [27,28,29]. The calibration curve was prepared by diluting hippuric acid standard solution into 50, 100, 250, 750, and 1000 mg/L with Millipore water. The sample preparation was conducted by mixing 0.5 mL urine sample with 0.5 ml pyridine and 0.2 ml benzene sulfonyl chloride in a polypropylene tube and then incubated at room temperature for 30 minutes. After the incubation, the mixture was then added 5 ml ethanol, centrifugated at 2000 rpm for 5 minutes, and absorbance was read at 410 nm. Analytical approach has accepted by Universitas Padjajaran, Indoneisa with references number 353/UN6.KEP/EC/2020.

### 2.6 Data analysis

Descriptive analysis was used to analyze respondent information and urinary hippuric acid concentration profile. Data normality was determined using the Shapiro-Wilk test. The comparison test between the exposed and control groups was done using the non-parametric statistic method Mann-Whitney, Kruskal-Wallis, and Chi-Square test, depending on the number of variables and types of data. Correlation analysis between urinary hippuric acid concentration and the numerical variable was done using Spearman correlation rank. The Odds ratio of each risk factor was determined using binary logistic regression analysis.

### 2.7 Ethical approval

The ethical clearance research was conducted closely between the parties involved in the research. The two-way agreement was obtained by verbal communication and signed a handwritten agreement. The Ethics Committee has approved this research of Padjajaran University, Bandung, Indonesia with No: 353/UN6.KEP/EC/2020.

## 3 Results and discussions

### 3.1 Respondent characteristics

A total of 30 respondents in the exposed group are dominated by age ranging 18–39 years old, while 22 respondents in the control group are dominated by 40–60 years old. Both groups are mainly non-smoker. Most of the exposed groups are light caffeine consumers (<3 glasses), while the control group is equal between light and heavy consumers. The caffeine consumption categories were made based on the study that reported a significant increase in uHA on heavy drinkers [11, 12, 30]. Most of the exposed groups are having a high school education level while the control group is elementary level. Except for age, all variables have no significant difference between the exposed and control group (Table 1). Age differences are caused by the residents who have an older age than those who are still actively working in the industry.

**Table 1:** Respondents’ characteristics on the exposed (N = 30) and the control group (N = 22).

Variable (n)	Exposed group n (%)	Control group n (%)
Gender		
Male	17 (56.67)	9 (40.91)
Female	13 (43.44)	13 (59.09)
Age (years)∗		
18–39	15 (50,00)	7 (26,92)
40–60	11 (36,67)	14 (53,85)
>60	4 (13,33)	5 (19,23)
Smoking habit		
Non-smoker	22 (73.33)	17 (77.27)
Smoker	8 (26.67)	5 (22.73)
Caffeine consumption^a^		
<3 glass	24 (80.00)	19 (86.36)
≥3 glasses	6 (20.00)	3 (13.64)
Education level		
Did not attend school	1 (3.33)	0 (0)
Elementary	6 (20.00)	11 (50.00)
Middle	6 (20.00)	4 (18.18)
High	16 (53.33)	5 (22.73)
University	1 (3.33)	2 (9.09)

∗statistically significant (p < 0,05).

a Caffeine in tea and coffee.

### 3.2 Working history

The exposed respondents are from the washing (43.33%), batik writing (40.00%), and batik stamping (16.67%) process (Table 2). The sample size of each process has been adjusted to the population distribution of each process. All workers worked for 8 hours a day, and most of them (83.33%) work 6 days a week and the rest work for 7 days a week. Most of the workers have been working for more than 2 years (73.33%). None of the workers work in the outdoor area. They work either in the indoor (36.67%) and semi-indoor room (63.33%). Although 93.33% workers said that mask is available in the working area, only 50% usually using mask while working. Workers said that using a mask is uncomfortable (36.67%) and even bothering their work (20%).

**Table 2:** Respondents’ characteristics based on working history.

Variable	Description	n	%
Working process	Writing *(Canting)*	12	40.00
	Stamping *(Cap)*	5	16.67
	Washing *(Celup*)∗	13	43.33
Working period	<2 years	8	26.67
	≥2 years	22	73.33
Working area	Indoor	11	36.67
	Semi-outdoor	19	63.33
Masker usage	Yes	10	33.33
	Sometimes	5	16.67
	No	15	50.00

∗ Washing including coloring, dyeing, and rinsing.

### 3.3 The urinary hippuric acid profile

The mean value of uHA in the exposed and control group are 420.91 and 387.99 mg/g creatinine, respectively (Table 3). For all respondents, the uHA concentration is below the recommended value set by The American Conference of Governmental Industrial Hygienists (ACGIH) that is < 1600 mg/g creatinine [31, 32]. In similar studies, high toluene exposure (mean 71.29 ppm) will escalate uHA concentration up to 5790 mg/g creatinine, way higher than the recommended value [33]. Meanwhile the low to medium toluene exposure (3–9 ppm) will significantly increase the uHA concentration (575–635 mg/g creatinine) although did not exceed the recommended value [16, 34]. In this study, the toluene concentration from preliminary data is categorized as a low concentration since it is below 1 ppm.

**Table 3:** Urinary hippuric acid profile on the exposed and control group.

uHA (mg/g creatinine)	Exposed (n = 30)	Control (n = 22)	p-value
Range	4.52–991.93	42.80–842.00	
Median	372.00	339.75	
Mean	420.91	387.99	0.566
Stdev	218.07	180.98	

Nevertheless, in this study, both groups have higher uHA mean and median concentration than background value without the toluene exposure and benzoic acid intake 180 mg/g [10]. This result is also higher than the uHA concentration that naturally produced by humans without toluene exposure, however the uncontrolled benzoic acid intake is 343 mg/g creatinine [9]. Although the control group also has a higher concentration than the background value, the increasing mean and median of uHA concentration on the exposed group compared to the control group showed that the exposed group has an excess toluene exposure that might be caused by occupational activities (Table 3). It should be noted that even though the difference and the uHA increase are not significant among the study groups, it does not mean that there is not an increase at all. The toluene exposure makes a difference on the uHA level but the magnitude of exposure that is in a low concentration has not elevated the uHA to the significant difference.

**Figure 1:**
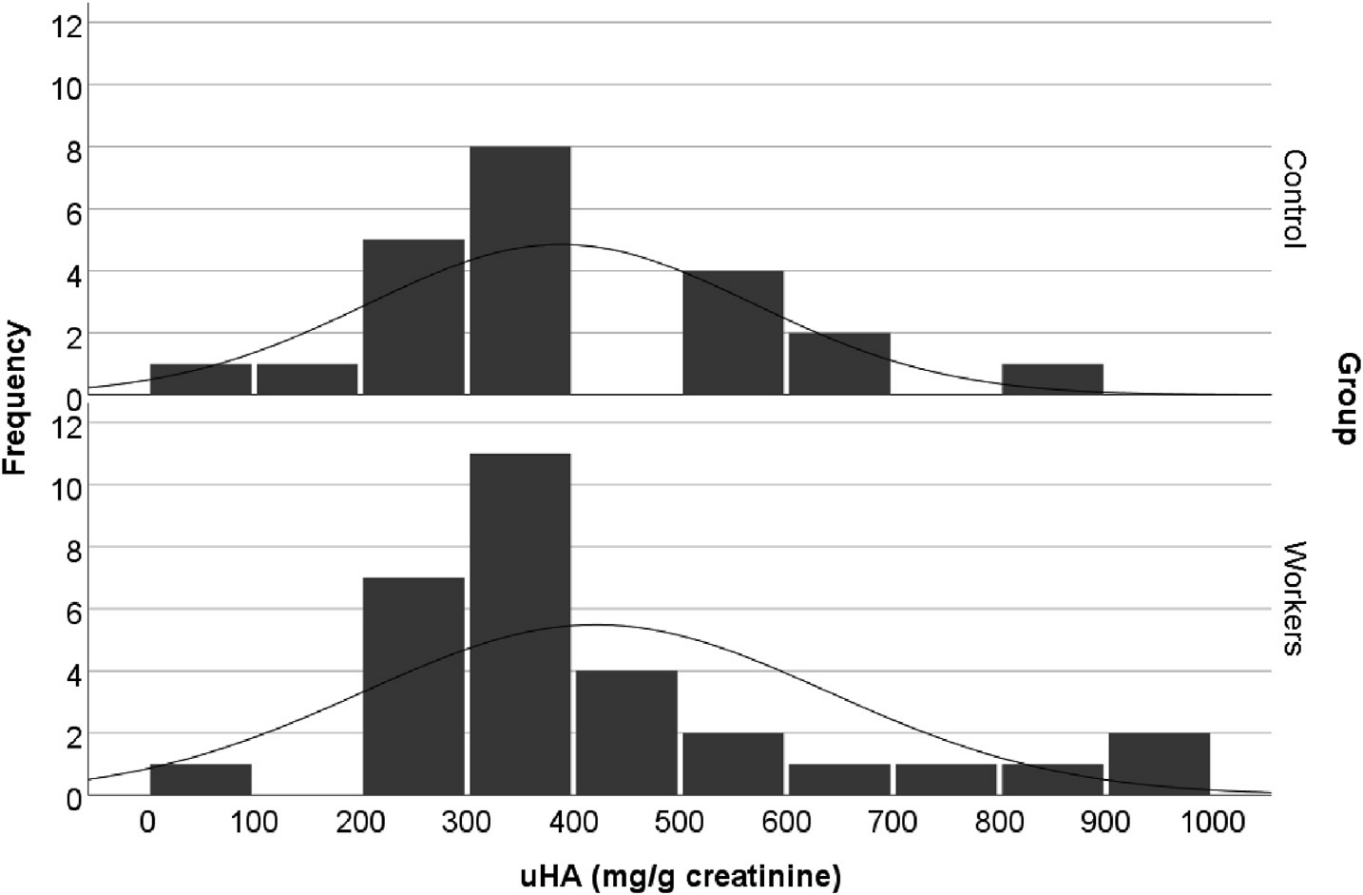
Distribution of uHA concentration on control and exposed group.

The uHA value distribution on the exposed group also is on the higher side with more than 200 up to 1000 mg/g creatinine compared to the control group that is clustered on the 200–700 mg/g creatinine (Figure 1). The increase of uHA in the control group was expected from consumption factors such as benzoic acid intake from food and drink preservatives in canned beverages, condiments (tomato and chili sauces), and instant noodle. However the total intake of these sources was not being controlled in this study. Berries family intake with high content of phenolic compound was excluded from the possibilities because of uncommon consumption in this study area [13].

We found an extreme value of uHA concentration on the control group with 842 mg/g creatinine. There are no unusual behaviors nor working histories on this respondent that could lead to be elevation of uHA. This respondent has a medical history with diabetic disease and takes medication routinely, but the medication does not alter the hippuric acid secretion. There should be a confounding factor affecting this result that could not be shown on the questionnaire result such as benzoic acid intake mentioned above.

In recent years, ACGIH suggests other metabolites to be used as a biomonitoring compound of toluene exposure, the ortho-cresol [31]. Ortho-cresol is a side product of toluene metabolism that has a specific feature to the toluene exposure. Ortho-cresol is not excreted naturally in humans' urinary content, so it can be used better to represent only toluene exposure. But, since this study toluene exposure is in a low concentration, and ortho-cresol only represents 5% of the absorbed toluene, it is feared that the ortho-cresol will also produce in a very low concentration and could not be detected. Other than that, ortho-cresol measurement is not common to use in Indonesia so there is a limitation in the instrumentation and measurement procedures.

Even so, based on the uHA concentration result which is difficult to specify the exact sources of uHA secretion due to the confounding factors in the consumption behavior, especially on the control group, other methods should be considered for further analysis. Whether using a specific biomarker such as urinary o-cresol and toluene or restrict the uHA sources intake of the respondents on the sampling day.

### 3.4 Factors associated with uHA concentration

Many factors have been associated with uHA concentration on humans. Individual factor such as gender, showing that adult female tends to produce uHA higher than males with the mean value respectively 200 mg/g creatinine and 160 mg/g creatinine [10]. The result of this current study supports the previous result by showing the uHA on the female worker is higher than in the male worker (Table 4). Smoking habits gave an inconsistent result on its correlation with uHA concentration. In this research, the non-smoker workers have a mean uHA concentration higher than the smoker. This result might be gender-associated since the non-smoker respondents, mostly female and female, have a higher concentration of uHA. The workers who consume more than 3 glasses of caffeine in tea or coffee have increased uHA concentration. This result was supported by other studies showing that caffeine intake significantly influencing the increase of uHA concentration [11, 12]. Tea and coffee consumption might give an additional amount of uHA produced by humans because of its high concentration of chlorogenic and quinic acid that can be metabolized into benzoic acid and enter the same pathway as a toluene metabolism into hippuric acid [12, 35].

**Table 4:** Comparison of uHA concentration on workers with certain risk factor.

Variable	n	Median uHA (mg/g creatinine)	Mean uHA (mg/g creatinine)	Range	p
**Gender**					
Male	17	353.52	395.54	4.52–883.14	0.464
Female	13	382.40	454.08	210.26–991.93	
**BMI**					
Not overweight	20	365.43	412.64	4.52–912.41	0.603
Overweight	10	372.00	440.20	210.26–991.93	
**Smoker**					
No	22	367.18	425.25	210.26–991.93	0.826
Yes	8	374.73	408.97	4.52–882.14	
**Caffeine consumption**					
Light (<3 glasses)	24	367.18	417.65	4.52–991.93	0.468
Heavy (≥3 glasses)	6	388.91	433.93	288.44–602.28	
**Working period**					
<2 years	8	309.96	281.31	4.52–448.03	0.035∗
≥2 years	22	387.12	471.68	216.19–991.93	
**Working process**					
Writing	12	421.83	512.79	210.26–991.93	0.096∗∗
Stamping	5	405.70	413.75	4.52–833.14	
Washing	13	331.48	388.85	216.19–602.28	

∗Statistically significant (p < 0.05) ∗∗ (p < 0.10) based on a non-parametric test.

**Figure 2:**
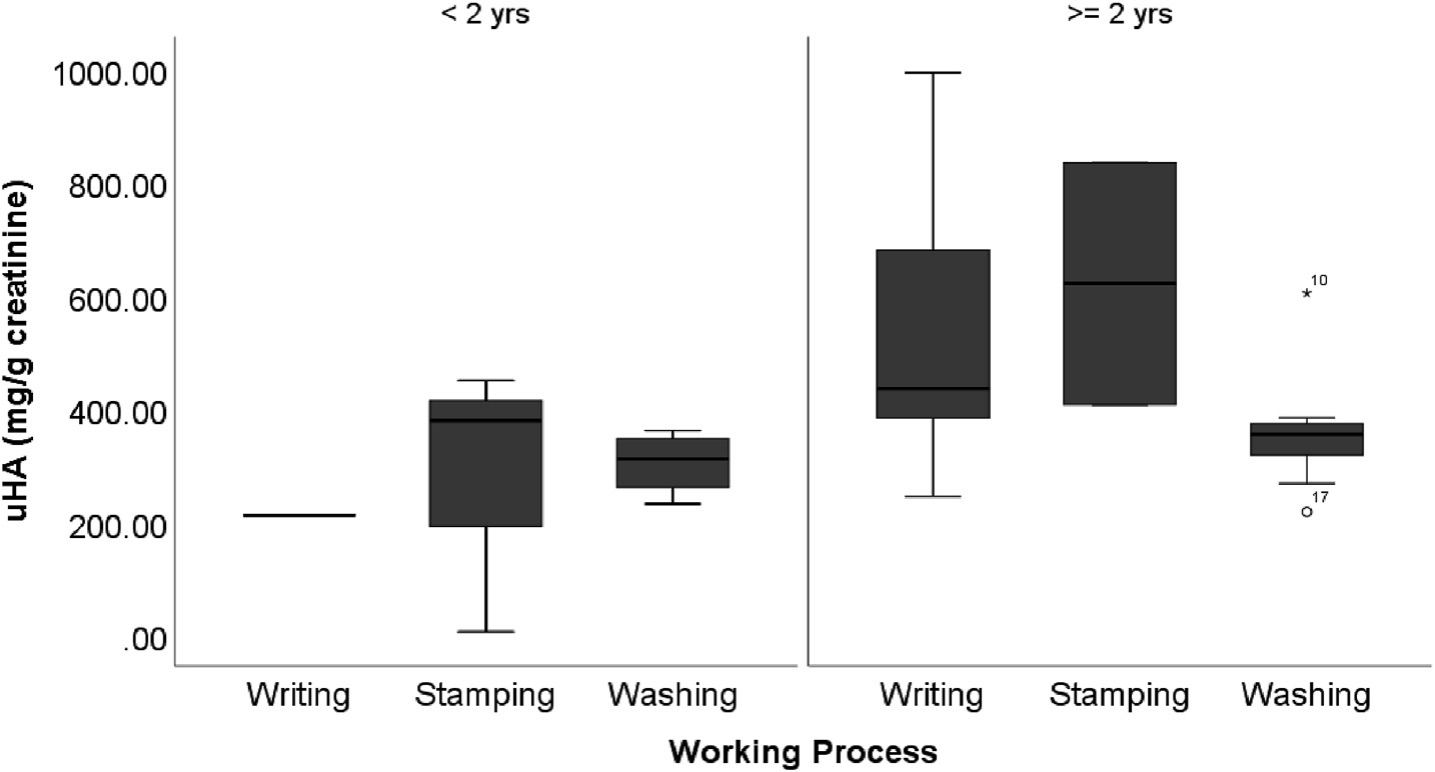
Comparison of uHA based on the working process and working experience.

Working period is the only variable that has a significant difference (p < 0,05). Workers with more than 2 years of experience are having a significant uHA concentration higher than workers with less than 2 years of experience (Table 4). When we tried to do a further grouping on uHA concentration based on the working process and working period, it showed that there is a significant increase in uHA concentration on writing and stamping workers with a working period of 2 years or more (see Figure 2).

The working period is related to repeated exposure and accumulation of toluene, which will be affecting CNS disease symptoms indicated by the higher level of excreted uHA. Based on the review by Filley (2004), neuropsychological symptoms caused by toluene exposure could be detected as early as 2 years after initial exposure, which corresponds with findings in this current study [36]. Comparison of uHA on working process showed that writing worker has the highest mean concentration followed by stamping worker and the last is washing worker. The writing and stamping process working with batik wax from other studies reported that fume exposure could increase batik workers' health problems [3, 4, 5]. A similar result with this study suggests that the batik wax usage workers had an excess exposure. One of them is from toluene exposure.

To determine the magnitude of probabilities on each risk factor on having a higher concentration of uHA, an Odds Ratio (OR) was calculated using binary logistic regression analysis. Female (OR 2.531), overweight based on BMI (OR 2.333), smoker (OR 1.389), and heavy caffeine consumer (OR 1.692) are factors that increase the probability of having a high level of uHA. But in this study, the elevation is statistically insignificant (p > 0,05). Working process and working period are the risk factors that have a significant increase in OR value. The writing process (OR 6.750) and workers with more than 2 years working period (OR 6.429) have a probability of having a higher level of uHA (p < 0,05). Stamping workers also having a higher probability with OR value of 9.000 but this group is statistically significant in different significance levels (p = 0,083) (Table 5).

**Table 5:** Odds Ratio (OR) of risk factors on increasing uHA level.

Variable (n = 52)	OR	p	95% CI
Age	0.991	0.974	0.952–1.048
Female	2.531	0.229	0.557–11.512
Overweight	2.333	0.303	0.466–11.693
Working period ≥2 years	6.429	0.047∗	0.141–8.995
Writing worker	6.750	0.033∗	1.162–39.200
Stamping worker	9.000	0.083∗∗	0.748–108.310
Smoker	1.389	0.698	0.264–7.299
Heavy caffeine consumer (≥3 glasses)	1.692	0.583	0.361–15.455

∗statistically significant (p < 0,05) ∗∗ (p < 0,10) using binary logistic regression.

The difference in process and materials used on writing and stamping workers suggested being a reason for a high concentration of uHA. Both working processes using batik wax that can generate toluene to the working area. In the working process, the batik wax was heated continuously throughout the working time, and the source of heat was near to the worker's body. The quantity of the usage of batik wax is also suggested as one of the factors. The stamping process uses stamping tools and a heating pan with a bigger surface area than on the writing process. The quantity of the fume generated and exposed to the workers might be higher in the stamping process.

### 3.5 Toluene exposure symptoms

The interviews regarding toluene exposure symptoms showed that most of the workers were having either acute or chronic symptoms. The highest percentage of toluene exposure symptoms experienced by batik workers are headache (50%) and insomnia (50%), followed by tiredness (40%), difficulty to concentrate (30%), nose irritation (27%), and loss of appetite (20%). Other symptoms such as nausea, eye irritation, and loss of smell were reported from less than 20% of workers.

Comparison of the impact of toluene exposure and uHA concentration showed that workers experiencing fatigue, insomnia, and loss of smell had higher mean uHA concentrations. Meanwhile, in acute toluene exposure assessment, the increasing uHA mean concentration was varied between workers with and without symptoms (Table 6). The assessment of the exposure symptoms could be confusing to conclude since it has a lot of confounding factors as it is not specific to one toxic chemical. The other exposure may induce similar symptoms. Nevertheless, this result is an important parameter as the symptoms are being experienced by workers and can be a direct sign that exposure happened.

**Table 6:** uHA concentration based on toluene exposure symptoms.

Effect	Symptoms	Mean uHA (mg/g creatinine)	
		No (n)	Yes (n)
Chronic	Tiredness	415.66 (18)	428.78 (12)∗
	Insomnia	409.94 (15)	431.88 (15)∗
	Loss of smell	408.37 (28)	596.54 (2)∗
	Difficult to concentrate	432.40 (21)	291.87 (9)
Acute	Headache	428.01 (15)	413.81 (15)
	Eye irritation	389.80 (26)	623.15 (4)∗
	Nausea	433.30 (26)	340.38 (4)
	Loss of appetite	444.80 (24)	325.34 (6)
	Respiratory irritation	430.29 (22)	395.11 (8)

∗ uHA with symptom is higher than without symptom.

### 3.6 Toluene exposure level

The uHA result showed that batik workers have a higher concentration on uHA than in the control group. It is also calculated that the working process and working period are the risk factor that significantly increases uHA in batik workers. To measure the level of toluene exposure, inhaled air sample was taken from each respondent, and toluene concentration was measured. In this study, the toluene measurement showing that the toluene content is in a very low concentration so that it could not be detected. The GC chromatogram showed a peak around the toluene retention area, but the intensity is very low, and the MS shows no significant result.

## 4 Conclusion

Batik workers have a high percentage of experiencing both acute and chronic toluene exposure symptoms. Workers with toluene exposure symptoms have a higher uHA concentration than workers without any symptoms. Toluene exposure monitored by uHA concentration showed that workers who have been working for more than 2 years are having a higher uHA concentration and probability 6.429 times higher on having a higher level of uHA. Workers who work using batik wax also have a high probability, 6.750 times and 9.000 times higher on having a higher level of uHA. The management of these risk factors should be considered in order to decrease the health risk on batik workers caused by occupational exposure.

## Declarations

### Author contribution statement

Katharina Oginawati: Conceived and designed the experiments; Performed the experiments.

Annisa Artsani Hanif Anka: Conceived and designed the experiments; Performed the experiments; Analyzed and interpreted the data; Contributed reagents, materials, analysis tools or data.

Septian Hadi Susetyo: Contributed reagents, materials, analysis tools or data; Wrote the paper.

Sri Awalia Febriana, Ikeu Tanziha, Cita Rosita Sigit Prakoeswa: Contributed reagents, materials, analysis tools or data.

### Funding statement

This work was supported by Kementerian Riset Teknologi Dan Pendidikan Tinggi Republik Indonesia, Riset Kolaborasi Indonesia (RKI) program.

### Data availability statement

Data will be made available on request.

### Declaration of interests statement

The authors declare no conflict of interest.

### Additional information

No additional information is available for this paper.
